# A novel technique for retrospective genetic analysis of the response to vaccination or infection using cell-free DNA from archived sheep serum and plasma

**DOI:** 10.1186/s13567-020-0737-9

**Published:** 2020-02-05

**Authors:** Eve Hanks, Helen Todd, Javier Palarea-Albaladejo, Tom N. McNeilly, Collette Britton, Keith T. Ballingall

**Affiliations:** 1 Institute of Biodiversity, Animal Health and Comparative Medicine, University of Glasgow, Bearsden Road, Glasgow, G61 1QH UK; 2 Moredun Research Institute, Pentlands Science Park, Penicuik, Edinburgh, EH26 0PZ UK; 3 Biomathematics and Statistics Scotland, JCMB, The King’s Buildings, Peter Guthrie Tait Road, Edinburgh, EH9 3FD UK; 4 SAC Consulting: Veterinary Services, SRUC Veterinary Services, Pentland Science Park, Bush Loan, Penicuik, Midlothian, EH26 0PZ UK

## Abstract

Genetic variation is associated with differences in disease resistance and susceptibility among individuals within a population. To date, molecular genetic analyses of host responses have relied on extraction of genomic DNA from whole blood or tissue samples. However, such samples are not routinely collected during large-scale field studies. We demonstrate that cell-free genomic DNA (cfDNA) may be extracted and amplified from archived plasma samples, allowing retrospective analysis of host genetic diversity. This technique was also applicable to archived serum samples up to 35 years old and to different ruminant species. As proof of concept, we used this cfDNA approach to genotype the major histocompatibility complex (MHC) class II *DRB1* locus of 224 Merino sheep which had participated in field trials of a commercial *Haemonchus contortus* vaccine, Barbervax^®^, in Australia. This identified a total of 51 different *DRB1* alleles and their relative frequencies. This is the first study to examine host MHC diversity using DNA extracted from archived plasma samples, an approach that may be applied to retrospective analyses of genetic diversity and responses to vaccination or infection across different species and populations.

## Introduction

Previous molecular studies of the influence of genetic diversity on the response to vaccination or infection have relied on the extraction of genomic DNA from blood or tissue samples collected during field trials. In the absence of such material, retrospective analyses are not easily achieved. However recent advances in the field of circulating cell free DNA (cfDNA) as potential biomarkers of disease [1, 2] led us to examine whether DNA of sufficient quality and quantity could be extracted from archived ovine plasma and serum samples for MHC genotyping. The re-use of material from previous vaccine trials allows implementation of the three Rs; replacement, reduction and refinement and, therefore, satisfies animal welfare and ethical use of animals in science requirements [3]. To demonstrate the utility of the method, we sought to determine whether genomic DNA could be obtained from archived plasma samples, and tested if alleles of the Ovar-MHC class II locus could be amplified from these samples. We focussed on the *DRB1* locus which is the most highly polymorphic MHC class II locus of domestic sheep (*Ovis aries*) [4, 5]. The MHC class II genes encode dimeric cell surface glycoproteins that present peptides to the adaptive immune system for recognition by CD4+ T cell receptors [6, 7]. MHC class II diversity plays an important role in determining the type and intensity of the immune response to foreign antigens within a population [4].

Previous studies have identified associations between MHC diversity and resistance or susceptibility to gastrointestinal nematodes (GIN) infection in different sheep breeds [8–12]. MHC influence on the response to GIN vaccination has not been examined. GIN infection is a major clinical and economic problem in humans and livestock and a key constraint to small ruminant productivity globally [13]. Current control relies on administration of anthelmintic drugs, however parasite resistance to the commonly used anthelmintics (benzimidazole, levamisole and avermectin classes) is widespread, with multidrug resistance having considerable impact in some areas [14]. The blood-feeding nematode *Haemonchus contortus* is the most pathogenic GIN of sheep and goats and the predominant species in warm climates, including large parts of Australia and South America [15, 16]. Barbervax^®^, the first vaccine commercialised for a GIN of any host, was launched in Australia in 2014 [17] and in South Africa in 2016 [18] after being developed in Scotland, UK. Vaccination is highly effective under controlled challenge conditions [> 70% reduction in worm burden and > 90% reduction in faecal egg count (FEC)] [19–21]. However under field challenge during vaccine trials, there was considerable variation between vaccinated individuals in their level of protection, as measured by faecal egg count, blood haemoglobin concentration and anti-Barbervax^®^ antibody titre [22].

In this retrospective analysis, we made use of archived plasma from Australian vaccine field trials to demonstrate the applicability of our novel PCR approach in detailing ovine MHC genotypes. While this study focussed on the MHC class II *DRB1* locus, the novel approach we describe using archived serum or plasma is equally applicable to retrospective studies of genetic diversity at other loci and to other infections or vaccinations.

## Materials and methods

### Plasma samples

Plasma samples were collected during 2011–2013 from Australian Merino sheep during nine Barbervax^®^ registration trials carried out by two companies; CSIRO and VHR, in New South Wales, Australia (Table 1). These studies were designed to test vaccine efficacy. Blood was obtained by jugular venepuncture using sodium heparin tubes and plasma separated at room temperature and stored at −20 °C. Sheep varied in age from 2 months to 2.5 years and included lambs, yearlings and ewes. The Australian Barbervax^®^ trials obtained ethical approval from the Australian Animal Ethics Committee for each trial and animals were handled in compliance with AEC and any applicable local regulations.

**Table 1:** Details of trials for Australian Merino sheep genotyped for this study

Company	Site	Year of trial	Number of sheep genotyped	Age of sheep
CSIRO	Armidale, New South Wales	2011	4	Lambs
CSIRO	Armidale, New South Wales	2012	30	Lambs
CSIRO	New South Wales	2012	27	Yearlings
CSIRO	New South Wales	2013	20	Ewes
VHR	Armidale, New South Wales	2012	36	Lambs
VHR	Dundee, New South Wales	2012	28	Yearlings
VHR	Kingston, New South Wales	2012	25	Yearlings
VHR	Dundee, New South Wales	2013	24	Ewes
VHR	Kingston, New South Wales	2013	30	Ewes

Serum was obtained from Texel cross sheep from a 2014 Barbervax^®^ trial at the Moredun Research Institute (MRI), with ethical approval from MRI Experiments and Ethics Committee (MRI E46 11) and were conducted under approved UK Home Office licence (PPL 60/03899) in accordance with the 1986 Animals (Scientific Procedures) Act. Ovine serum collected between 1984 and 2004 and archived bovine and caprine serum were obtained from MRI archives.

### Barbervax^®^ vaccination schedules

Merino lambs were vaccinated subcutaneously five times during the grazing season over spring and summer, and yearlings and ewes given four doses of vaccine at 3–6 weekly intervals. Blood was sampled from all sheep on vaccination days or at fortnightly intervals for anti-Barbervax^®^ Ab titre measured by ELISA, as described previously [23]. Sheep were organised by trial location, year and age, as shown in Table 1, and totalled 9 separate trials from 2011 to 2013. In each trial 20–40 animals were vaccinated with Barbervax^®^ from which 224 sheep were genotyped.

### Preparation of genomic DNA

Genomic DNA (gDNA) was prepared in the UK, from archived plasma samples using the Qiagen DNeasy Blood & Tissue kit (Qiagen UK, 69504), with the only modification to the standard procedure being the use of serum or plasma instead of blood or tissue. The following was added to a 1.5 mL Eppendorf tube: 100 µL serum or plasma, 100 µL PBS, 20 µL Proteinase K and 200 µL of buffer AL. The protocol provided with the kit was then followed. Eluted DNA (50 µL) was quantified using Nanodrop spectrophotometry (ThermoFisher Scientific). This indicated a typical concentration of 5–15 ng/µL, therefore a yield of 250–750 ng gDNA per 100 µL plasma was obtained. There was no obvious difference in yield of gDNA using serum or plasma.

### PCR amplification of the ovine MHC class II *DRB1* locus

Oligonucleotide PCR primers targeting the 5′ and 3′ intronic regions flanking, or partially overlapping the highly polymorphic second exon of the *Ovar*-*DRB1* gene were utilised. Primers are listed in Table 2 and their locations relative to the second exon of the *DRB1* gene are shown diagrammatically in Additional file 1. The primers had previously been designed to amplify *DRB1* exon 2 using DNA from whole blood [6], with the exception of nested primers 455 and KBEH1 which were new to this study. Different primer combinations were used in nested or hemi-nested PCR reactions to optimise amplification of the second exon using the DNA isolated from plasma. Primers 275 and 329 were used in the first round PCR then 455 and 329 in the second round or, if no amplification was achieved, an alternative combination of PCR primers, 330 and 329 in the first round and 455 and 329 in the second round were used. The combination of primers 455 and 329 in the first round and primers KBEH1 and 329 in the second round improved the amplification of both alleles in animals initially typed as homozygous and this primer combination was used to validate all homozygous animals.

**Table 2:** Oligonucleotide primers used for amplification of the second exon of the *Ovar*-*DRB1* locus. Adapted from [6]

Primer name	Position relative to the first base of the second exon of the *Ovar*-*DRB1* locus	Forward (F) or reverse (R)	Sequence
275	Intron 1 (−55 to −35)	F	ATTAGCCTCTCCCCAGGAGTC
329	Exon 2/intron 2 (263 to +15)	R	CACCCCCGCGCTCAC/CTCGCCGC
330	Intron 1 (−55 to −35)	F	ATTAGCCTCYCCCCAGGAGKC
455	Intron 1/exon 2 (−16 to +8)	F	TATCCCGTCTCTGCAG/CACATTTC
KBEH1	Intron 1/exon 2 (−7 to +14)	F	TCTGCAG/CACATTTCYTGGAG

Intron 1 sequence is indicated as negative, intron 2 sequence is indicated as positive. Positions 1 to 270 indicate exon 2. The intron exon boundary is marked by /.

Nested and hemi-nested PCR was conducted in 50 µL reactions consisting of 200 nM of each primer, 1 Unit One*Taq* polymerase (New England BioLabs, M0480S) and corresponding buffer and 2–4 µL (20–40 ng) of purified gDNA as template. Two microliters of first round PCR product was used as template in a second round of PCR. First round PCR cycling conditions were: 94 °C for 2 min, followed by 15–20 cycles at 94 °C for 30 s, 58–60 °C for 30 s, and 72 °C for 30 s, with a final extension at 72 °C for 4 min. The second round used the same cycling conditions, but for 30 cycles. PCR products were analysed by electrophoresis on 1% agarose gels and sent directly for purification and sequencing or sequenced following gel excision and purification using the Wizard SV Gel and PCR Clean-Up System (Promega, A9281). All PCR products were sequenced in both directions by Eurofins Genetic Services (Germany), using terminal primers.

### Cloning of *Ovar*-*DRB1* PCR products

Alleles that proved difficult to annotate from direct sequencing of the PCR product were cloned into the pGEM-T-easy vector (Promega, A1360) and transformed into *E. coli* JM109 cells, applying blue/white selection with IPTG (isopropyl β-d-1-thiogalatopyranoside, Merck 367-93-1) and X-gal (5-bromo-4-chloro-3-indolyl β-d-galactopyranoside, ThermoFisher Scientific B1690). Twelve colonies per PCR product were PCR screened for the correct insert using primers 455 and 329. Individual clones representing both alleles were identified by digestion of the colony PCR product with 5 U of the frequent cutting restriction enzyme RsaI (ThermoFisher Scientific, ER1121) at 37 °C for 1 h [6]. RsaI was inactivated at 80 °C and digested PCR products (15 µL) were separated on an 8% polyacrylamide gel. DNA from a minimum of three clones representing both alleles, as indicated by the RsaI digest pattern, was sequenced in both directions.

### *Ovar*-*DRB1* sequence analysis

Sequences were analysed using the SeqMan Pro 14 program within the DNASTAR software package. Contigs were assembled from the forward and reverse DNA sequences from each plasma sample. Heterozygous positions were manually labelled according to the International Union of Biochemistry (IUB) ambiguity code (Nomenclature Committee of the International Union of Biochemistry, 1984). Each sequence was searched against a database of all known *Ovar*-*DRB1* alleles downloaded from the immunopolymorphism database (IPD)-MHC database [5, 7, 24] using a Basic Local Alignment Search Tool (BLAST). The two alleles providing the highest alignment scores were then manually checked to ensure a perfect match.

## Results

### Amplification of MHC class II *DRB1* alleles from archived plasma from Merino sheep

The second exon of the ovine MHC class II *DRB1* locus was successfully amplified by hemi-nested PCR from DNA template extracted from archived plasma samples from Merino sheep. The samples had been collected during 2011–2013 Australian Barbervax field trials and stored at −20 °C prior to DNA extraction. From a total of 257 samples initially tested by PCR, products were obtained from 224 Merino samples (87%). This demonstrated that DNA of sufficient quantity and quality can be extracted from most archived plasma samples for amplification of the *DRB1* locus.

### Sequence analysis of ovine *DRB1* alleles

Two hundred and twenty-four DNA samples provided sequence of a quality that allowed identification of one or both alleles directly from the PCR product. Within this, thirty-two sequences obtained from these 224 samples (7%) were of insufficient quality to determine allele type directly. This was resolved by cloning the PCR products from these samples into the pGEM-T-Easy vector and sequencing the cloned products (see “Materials and methods”).

All alleles identified in this study were present within the IPD-MHC Database [24]. In total, 51 alleles were identified. Of the 224 sheep genotyped at the *DRB1* locus, 14 (frequency of 0.063) were homozygous. This value is similar to previous reports across a range of sheep breeds (0.075) [6]. The 7 most frequently occurring alleles are listed in Table 3 and the remaining 44 alleles identified and their frequencies are shown in Additional file 2.

**Table 3:** Relative frequency of the seven most abundant *Ovar*-*DRB1* alleles in Merino sheep

Allele name	Total	Relative frequency
04:01	21	0.050
07:01	37	0.088
08:03	48	0.114
15:02	46	0.109
17:02	20	0.048
19:02	28	0.067
20:02	29	0.069

### Application to other breeds, species and samples of different ages

Additionally, sera from seven Texel cross sheep, from a 2014 Barbervax^®^ trial at the Moredun Research Institute (MRI), UK, were PCR amplified and sequenced. This demonstrated that the technique can be applied across different breeds (representative PCR data shown in Additional file 3). To test application of the approach to older samples and to different species, we also included three ovine serum samples from the MRI archive from 1984, three from 1994 and three from 2004. In addition, we tested amplification using three bovine and three caprine serum samples provided by the Virus Surveillance Unit at MRI. All samples produced PCR products of the expected size.

## Discussion

MHC genotype can impact on host responses to parasitic disease by influencing the range of antigenic peptides presented for recognition by the adaptive immune system. Numerous studies have shown the relevance of MHC diversity on resistance or susceptibility to infection with nematode parasites in many species including mice [25, 26], sheep [8–12] and cattle [27]. However, MHC genotyping has relied on the availability of cell or tissue samples for DNA extraction. Here we report for the first time that plasma or serum may be employed as an appropriate source of genomic DNA for sequence based analysis of MHC allelic diversity. Our high success rate of amplification and sequencing shows that the yield and quality of DNA obtained from archived plasma or serum samples is sufficient for detailed, retrospective analysis of MHC diversity in population based studies. Using this approach we were able to genotype the MHC *DRB1* locus of 224 sheep and differentiate between homozygous and heterozygous animals. The method we developed is relevant to future monitoring of genetic influences on vaccine efficacy, disease susceptibility and to selective breeding. Additionally, it opens up the opportunity to include targeted DNA sequence analysis retrospectively to studies that have relied on phenotypic data alone. The approach is also amenable to high throughout sequencing of pooled amplicons from of large numbers of samples using molecular barcodes incorporated into each primer.

The source of DNA present in the serum or plasma samples is currently unknown. It seems unlikely to be derived from lysed white blood cells as the samples showed no evidence of cell lysis and the amount of DNA extracted was consistent across all samples. The most likely source is cfDNA. cfDNA was first discovered in 1948 in human plasma [28] and is now known to be released by a variety of cells into the circulation [29]. It has been shown that tissue damage such as cardiomyocyte death [30], inflammatory processes such as venous thromboembolism [31] and potentially parasitic infections [32], may all be characterised by detection of cfDNA. This technique is non-invasive, apart from the initial blood sampling, and the use of cfDNA for biomarker discovery is rapidly increasing, particularly for diagnosis, monitoring and prognosis of specific cancers; a “liquid biopsy” [33]. Furthermore, cfDNA may be biologically active and has been shown to have immunoregulatory effects in macrophages in vitro [34].

Here, to demonstrate application of the technique, we focused on amplification of the highly polymorphic second exon of the MHC class II *DRB1* gene in sheep, the major region encoding the peptide-antigen binding site [35]. However, this approach is equally applicable to other loci that may influence or be used as markers of response to vaccination or infection. Having established the technique, it will be important to determine its wider applicability to disease monitoring, diagnostics and selective breeding. We successfully amplified and sequenced the *DRB1* locus from 87% (224/257) of plasma samples from Australian field trials of the Barbervax^®^ vaccine. No novel *DRB1* alleles were identified, which is both a testament to the comprehensive nature of the IPD-MHC database and the reliability of the approach. Contamination is a potential pitfall associated with all PCR amplification techniques. We had no evidence of contamination and no disparity in the number of homozygous animals in comparison to similar studies using DNA extracted from whole blood (“Sequence analysis of ovine DRB1 alleles” section) and no evidence that one or two alleles dominated the findings.

Optimisation of the PCR procedure by employing different combinations of primers allowed reliable identification of animals homozygous or heterozygous at the *DRB1* locus. Homozygosity was represented by only a small number of sheep (14). Current theory suggests that the evolution and maintenance of MHC diversity is likely to be driven by interaction with rapidly evolving pathogens and that heterozygosity offers some advantage in protection against infection [10, 36, 37]. It is worth noting that homozygosity at the *DRB1* locus does not mean that the animal is always homozygous across the MHC class II region. The ovine MHC class II region includes a range of polymorphic *DQ* loci [38] and a number of studies have identified diversity at the duplicated *DQA* and *DQB* loci linked with identical *DRB1* alleles [4].

A total of 224 Merino sheep were successfully genotyped at the *DRB1* locus. Fifty-one alleles were identified at relative frequencies ranging from 0.114 to 0.002; the level of heterozygosity supports the concept that all available alleles were amplified. The same PCR primer combinations amplified *DRB1* from DNA isolated from the serum of Texel cross sheep and from bovine and caprine serum, demonstrating the wide applicability of the technique, although primer optimisation may be required for each species. In previous studies, the primer combinations employed in this study successfully amplified *DRB1* from genomic DNA obtained from whole blood samples from a range of sheep breeds [6] and from goats [39, 40]. In this study, amplification of the *Ovar*-*DRB1* locus failed in 33 samples. This is most likely due to degradation of the DNA following storage and freeze/thaw cycles rather than a failure to amplify certain alleles.

No specific *DRB1* alleles have previously been associated with resistance to *H. contortus* infection although microsatellite [10] and single nucleotide polymorphisms within *Ovar*-*DRB1* have been identified in resistant and susceptible sheep from St. Croix, Katahdin and Dorper breeds [12]. One *DRB1* allele (11:01) has been associated in Suffolk sheep with lower worm burdens and higher mast cell and plasma platelet counts following infection with the GIN *Teladorsagia circumcincta* [36] and with reduced faecal egg count in Texel breed sheep [41]. In future vaccine studies, where more comprehensive information is available, such as animal pedigree, formal statistical generalised linear mixed modelling (GLMM) may be applied to determine any associations between MHC genotype and vaccine efficacy, to better understand host protective responses to vaccination and infection.

In summary, this is the first report of DNA extraction and amplification from plasma or sera for retrospective use in genetic analysis of the outcome of a vaccine field trial. Moreover, we routinely extracted good quality DNA from samples stored for many years, demonstrating a new route to accessing genetic information from archived samples that can be widely applied to examine the effects of MHC and other genes on the outcome of parasitic infections. While this study focussed on the MHC class II *DRB1* locus, the approach is equally applicable to retrospective studies of genetic diversity at other loci.

## Supplementary information

**Additional file 1. Diagram of*Ovar*-*DRB1*Exon 2 region showing the position of each primer used in PCR genotyping.**

**Additional file 2. Low frequency*Ovar*-*DRB1*alleles sequenced from Merino sheep.**

**Additional file 3. Specific PCR amplification of** ***Ovar*-*DRB*-*1*** **exon 2 using hemi-nested PCR.** Agarose gel electrophoresis of PCR products of DNA from plasma of seven Merinos lambs (1–7). Negative control PCR (N, no template DNA) showed no amplified product. Mr indicates 100 bp DNA ladder.

## Abbreviations

MHC: major histocompatibility complex
GIN: gastrointestinal nematode
cfDNA: cell-free DNA
gDNA: genomic DNA
IPD: immunopolymorphism database
BLAST: Basic Local Alignment Search Tool
