# Supplementary figures and images for: A novel technique for retrospective genetic analysis of the response to vaccination or infection using cell-free DNA from archived sheep serum and plasma
Source: Vet Res. 2020 Feb 5;51:9. doi: 10.1186/s13567-020-0737-9 (PMC7003321; doi:10.1186/s13567-020-0737-9)

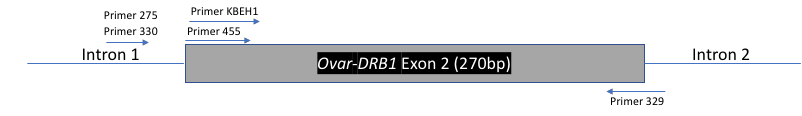

Supplement: Supplementary file 1 — Additional file 1. Diagram ofOvar-DRB1Exon 2 region showing the position of each primer used in PCR genotyping. [file 13567_2020_737_MOESM1_ESM.docx]
