# Supplementary material for: A novel technique for retrospective genetic analysis of the response to vaccination or infection using cell-free DNA from archived sheep serum and plasma
Source: Vet Res. 2020 Feb 5;51:9. doi: 10.1186/s13567-020-0737-9 (PMC7003321; doi:10.1186/s13567-020-0737-9)
Supplement: Supplementary file 2 — Additional file 2. Low frequencyOvar-DRB1alleles sequenced from Merino sheep. [file 13567_2020_737_MOESM2_ESM.docx]

| **Allele Name** | **Relative frequency** |
| --- | --- |
| 03:08 | 0.038 |
| 04:02 | 0.034 |
| 10:01 | 0.036 |
| 16:05 | 0.036 |
| 19:03 | 0.033 |
| 19:01 | 0.033 |
| 07:02 | 0.024 |
| 20:01 | 0.024 |
| 16:04 | 0.021 |
| 08:01 | 0.017 |
| 07:04 | 0.012 |
| 03:09 | 0.012 |
| 09:01 | 0.012 |
| 08:02 | 0.010 |
| 21:01 | 0.010 |
| 01:03 | 0.010 |
| 03:03 | 0.007 |
| 16:08 | 0.007 |
| 10:05 | 0.005 |
| 03:10 | 0.005 |
| 03:01 | 0.005 |
| 16:02 | 0.005 |
| 10:06 | 0.005 |
| 08:04 | 0.002 |
| 12:04 | 0.002 |
| 22:02 | 0.002 |
| 12:02 | 0.002 |
| 12:01 | 0.002 |
| 10:02 | 0.002 |
| 20:03 | 0.002 |
| 08:05 | 0.002 |
| 22:01 | 0.002 |
| 02:01 | 0.002 |
| 01:04 | 0.002 |
| 05:01 | 0.002 |
| 15:01 | 0.002 |
| 17:01 | 0.002 |
| 15:03 | 0.002 |
| 08:06 | 0.002 |
| 13:01 | 0.002 |
| 04:03 | 0.002 |
| 24:01 | 0.002 |
| 01:01 | 0.002 |
| 09:02 | 0.002 |
