# Supplementary material for: A novel technique for retrospective genetic analysis of the response to vaccination or infection using cell-free DNA from archived sheep serum and plasma
Source: Vet Res. 2020 Feb 5;51:9. doi: 10.1186/s13567-020-0737-9 (PMC7003321; doi:10.1186/s13567-020-0737-9)
Supplement: Supplementary file 3 [file 13567_2020_737_MOESM3_ESM.docx]

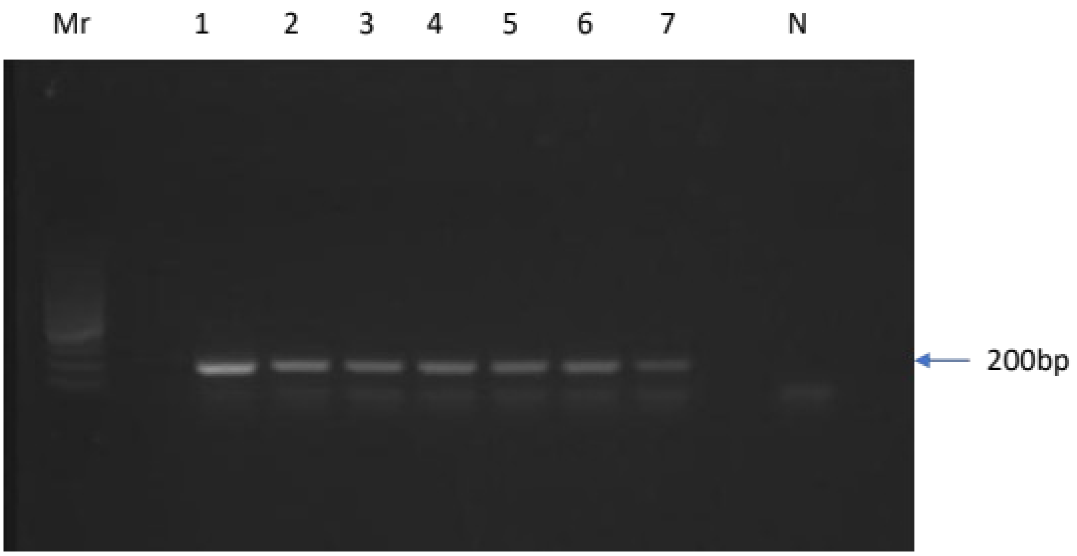
Mr
1
2
3
4
5
6
7
N
200bp
